# The 2018 new definition of periprosthetic joint infection improves the diagnostic efficiency in the Chinese population

**DOI:** 10.1186/s13018-019-1185-y

**Published:** 2019-05-24

**Authors:** Haitao Guan, Jun Fu, Xiang Li, Wei Chai, Libo Hao, Rui Li, Jing Zhao, Jiying Chen

**Affiliations:** 1 Department of Orthopaedics, Chinese People’s Liberation Army General Hospital (301 Hospital), Beijing, People’s Republic of China; 2 Anesthesia/Surgery Center, Chinese People’s Liberation Army General Hospital (301 Hospital), Beijing, People’s Republic of China

**Keywords:** Periprosthetic joint infection, Diagnosis, New definition, Chinese population

## Abstract

**Background:**

Periprosthetic joint infection (PJI) is a devastating complication following total joint arthroplasty (TJA). Now, the definition of PJI traditionally used in clinical practice was set out by the International Consensus Meeting (ICM) and Infectious Diseases Society (IDSA). There was a new definition proposed in May 2018 on a paper published in the Journal of Arthroplasty. The new scoring system for PJI demonstrated a higher sensitivity and specificity than Musculoskeletal Infection Society and IDSA criteria. Therefore, we wanted to find further evidence to support the new definition in the Chinese population.

**Methods:**

The patients who were included in our study were divided into PJI group and aseptic group. Medical records of patients (98 in PJI group and 165 in aseptic group) were reviewed, and the score of every patient was aggregated based on the new definition and collected data. The sensitivity and specificity were compared between new definition and classical criteria.

**Results:**

For patients in our hospital, the overall sensitivity and specificity of the new criteria were respectively 94.9% (95% confidence interval [CI] 87.9–98.1%) and 95.2% (95% CI 90.3–97.7%). The new definition demonstrated a higher sensitivity than traditional criteria in Chinese population, and the specificity was similar to existing criteria.

**Conclusion:**

We believe the new scoring system about periprosthetic joint infection could also apply to Chinese population for diagnosing PJI following TJA. It can obviously improve diagnostic efficiency for PJI compared with traditional criteria.

## Introduction

Periprosthetic joint infection (PJI) is the most serious complication that lowers the quality of patients’ life by elevating the patients’ burden of cost and affecting the outcome of total joint arthroplasty (TJA) [1–3]. Delanois et al. reported in 2017 that infection was the most common etiology for revision TKA (20.4%) in the USA [4]. Therefore, PJI causes major concern for surgeons and patients. Meanwhile, a large amount of advanced means of diagnosis and treatment were introduced and even applied clinically [5–7]. However, there is no agreement on the definition of PJI that has been reached, and new diagnostic criteria are proposed constantly for diagnosing PJI more accurately [8–12]. Among these criteria, the definitions standardized by the Musculoskeletal Infection Society (MSIS) and the Infectious Diseases Society (IDSA) are widely accepted by researchers and surgeons [8, 9]. The MSIS criteria were partly revised in some diagnostic indicator at International Consensus Meeting (ICM) in 2013 and then more widely used in clinical practice [10].

In recent years, some new markers are utilized clinically and proved to be useful. Researchers evaluated serum markers, synovial markers, and intraoperative findings and finally proposed an evidence-based, weight-adjusted scoring system for definition of PJI in hip and knee in 2018 [13]. The new definition was then validated on an external cohort of 222 patients with PJI and 200 aseptic patients. The new criteria demonstrated a higher sensitivity of 97.7% compared to the MSIS (79.3%) and ICM definition (86.9%), with a similar specificity of 99.5% [13].

The purpose of our study was to validate the new criteria on patients that underwent revision THA and TKA in our hospital for PJI or aseptic loosening. Then, the new criteria’s performance on our cohort will be compared with that of original research and new criteria will be evaluated whether can be applied on every occasion.

## Materials and methods

We conducted a retrospective review and collected information of all patients who underwent revision total hip (THA) and knee (TKA) arthroplasty from our hospital between January 2015 and August 2017. Patients who had been placed antibiotic-loaded cement spacer in their joint at admission, had long-time antibiotic history before surgery, and had multiple history of surgery were also excluded.

### Patient population

We validated the new criteria on patients that underwent revision THA and TKA in the Chinese PLA General Hospital for PJI or aseptic loosening. All patients included in our study were divided into two groups according to strict filtering criteria: PJI cases and aseptic cases.

- PJI cases: Because there were no difference in major criteria between new criteria and MSIS and ICM criteria, we regarded the identical major criteria as “gold standard” of PJI. A patient was considered as a PJI case if he or she met the major criteria of MSIS, ICM, and new criteria. Therefore, the patients who had a presence of a sinus tract communicated to articular cavity or two positive cultures isolating the same pathogen from intraoperative periprosthetic tissue or synovial fluid samples through aspiration were included in the PJI group. In reality, we just compared the minor criteria between the new definition and ICM and IDSA criteria. Data from the first infection was documented.
- Aseptic cases: This group was composed of patients who were regarded underwent aseptic revision. Aseptic revisions were defined as cases undergoing one-stage revisions for the reasons except for infection. Meanwhile, the one-stage revisions did not fail because of infection and the aseptic cases never underwent a surgery for infection again on the same joint with more than 1-year follow-ups.

### Data collection

Patients’ basic information such as characteristics, comorbidities, and surgery information were collected first through reviewing patients’ medical records. Then, the diagnostic information, including presence of sinus tract, positive culture, laboratory results (serum, synovial), and intraoperative findings (purulence and histopathology), were documented. Based on the new scoring system, preoperative and intraoperative diagnostic scores were calculated and patients were “diagnosed” of PJI, aseptic loosening, or inconclusive cases according to the new diagnostic criteria. The patients having an aggregated score of greater than or equal to 6 are considered as infected, and a score of 3 or less represents not infected. The patients who are scored between 4 and 5 could not be diagnosed of PJI or aseptic [13]. Patients’ preoperative and intraoperative information will also be evaluated based on criteria of ICM and IDSA.

International Consensus Meeting (ICM) criteria agreed that PJI exists when:

1. Two positive periprosthetic cultures with phenotypically identical organisms
2. A sinus tract communicating with the joint
3. Three of the following six criteria exist:
  - Elevated serum C-reactive protein (CRP) AND erythrocyte sedimentation rate (ESR)
  - A single positive culture
  - Elevated synovial fluid white blood cell (WBC) count
  - ++ change on leukocyte esterase test strip
  - Elevated synovial fluid polymorphonuclear neutrophil percentage (PMN%)
  - Positive histological analysis of periprosthetic tissue [10]

According to the Infectious Diseases Society of America (IDSA), the definition of PJI is:

1. The presence of a sinus tract that communicates with the prosthesis.
2. The presence of acute inflammation based on histopathologic examination of periprosthetic tissue at the time of surgical debridement or prosthesis removal.
3. The presence of purulence surrounding the prosthesis.
4. Two or more intraoperative cultures or combination of preoperative aspiration and intraoperative cultures that yield the same organism. Growth of a virulent microorganism (e.g., *Staphylococcus aureus*) in a single specimen of a tissue biopsy or synovial fluid may also represent PJI.
5. The presence of PJI is possible even if the above criteria are not met; the clinician should use his/her clinical judgment to determine if this is the case after reviewing all the available preoperative and intraoperative information [8].

New scoring-based definition for periprosthetic joint infection (PJI) is presented in Table 1 [13], and the sensitivity and specificity will finally be compared among various criteria.

**Table 1:**
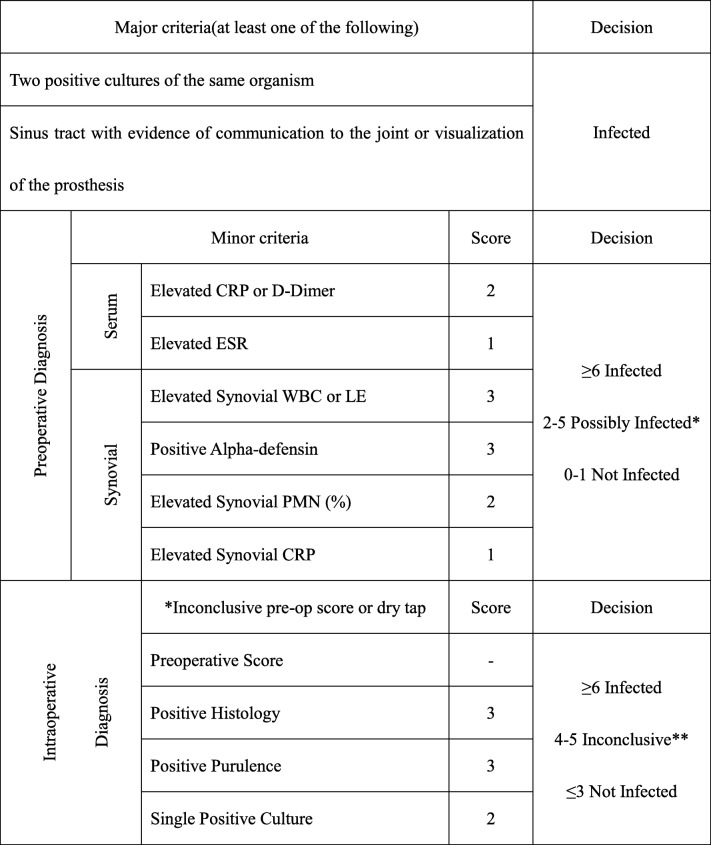
New scoring based definition for periprosthetic joint infection (PJI)

Proceed with caution in: adverse local tissue reaction, crystal deposition disease, slow growing organisms

*For patients with inconclusive minor criteria, operative criteria can also be used to fulfill definition for PJI

**Consider further molecular diagnostics such as next-generation sequencing

### Statistical analysis

All statistical analyses were performed using IBM SPSS statistic version 20 (SPSS Inc., Armonk, NY). For all sites, descriptive analyses were performed to calculate means, standard deviations (±), and frequencies (%). To evaluate any differences in diagnostic accuracy among various criteria, true positives, true negatives, false positives, false negatives, positive predictive value, and negative predictive value were calculated. The sensitivity and specificity with 95% confidence intervals (CI) will finally be compared among various criteria. Student’s *t* test and chi-square test were used to compare demographics and clinical characteristics between PJI and aseptic group. Statistical significance was defined as *P* value < 0.05.

## Results

Overall patients treated with revision surgery in our hospital between January 2015 and August 2017 were finally included in our study. Based on the filtering criteria as the above, 98 of them were included in the PJI cohort and 165 were included in the aseptic cohort. We retrospectively reviewed demographics and clinical characteristics of these patients such as age, gender, race, joint, information of surgery, and comorbidities. Patients’ demographic details and basic information about medical history are outlined in Table 2. Patients in the PJI and aseptic cohorts were all graded based on the new criteria, and the aggregated scores which consist of preoperative and intraoperative scores would be used to evaluate whether patients were infected. Of the 98 patients in the PJI cohort, 78 (79.6%) were finally diagnosed as infected according to the new criteria, 5 (5.1%) were falsely diagnosed as not infected, and 15 (15.3%) could not be diagnosed definitively. In the aseptic group, 145 (87.9%) patients were correctly regarded as not infected and 8 (4.8%) were falsely diagnosed as PJI cases, and for the rest 12 (7.3%) cases, accurate judgements could not be made based on the new criteria. In conclusion, for patients in our hospital, the overall sensitivity and specificity of the new criteria were respectively 94.9% (95% confidence interval [CI] 87.9–98.1%) and 95.2% (95% CI 90.3–97.7%). The positive predictive value (PV+) and negative predictive value (PV−) of the new criteria were respectively 92.1% (95% CI 84.5–96.3%) and 96.9% (95% [CI] 92.6–98.9%). (Table 3).

**Table 2:** Demographics of patients in PJI cohort (*n* = 98) and aseptic cohort (*n* = 165)

Variable	Overall (*n* = 263)	PJI cohort (*n* = 98)	Aseptic cohort (*n* = 165)	*p* value
Age	61.4 (12.1)	63.1 (13.5)	60.4 (14.1)	0.084
Gender (male)	116 (44.1%)	47 (48.0%)	69 (41.8%)	0.332
Joint (knee)	75 (28.5%)	40 (40.8%)	35 (21.2%)	0.001^*^
Time from last surgery (yr)	8.4 (5.9)	4.1 (4.5)	10.2 (6.8)	< 0.001^*^
Most recent surgery a revision procedure	51 (19.4%)	27 (27.6%)	24 (14.5%)	0.01^*^
History of rheumatoid arthritis and ankylosing spondylitis	24 (9.1%)	5 (5.1%)	19 (11.5%)	0.081
History of malignancy	12 (4.6%)	3 (3.1%)	9 (5.5%)	0.368
History of diabetes	39 (14.8%)	21 (21.4%)	18 (10.9%)	0.02^*^

Quantitative data is presented as mean (standard deviation). Qualitative data is presented as number (%)

*yr* year

*p < 0.05: statistically significant

**Table 3:** The comparison of diagnostic outcome between the new criteria and the classical criteria by the International Consensus Meeting (ICM) and Infectious Diseases Society of America (IDSA)

	PJI cohort (*n* = 98)			Aseptic cohort (*n* = 165)			Sensitivity (95% CI)	Specificity (95% CI)	PV^+^ (95% CI)	PV^−^ (95% CI)
	True positives	False negatives	Inconclusive	True negative	False positives	Inconclusive				
New criteria	78 (79.6%)	5 (5.1%)	15 (15.3%)	145 (87.9%)	8 (4.8%)	12 (7.3%)	94.9% (87.9–98.1%)	95.2% (90.3–97.7%)	92.1% (84.5–96.3%)	96.9% (92.6–98.9%)
ICM (2013)	52 (53.1%)	46 (46.9%)	/	163 (98.8%)	2 (1.2%)	/	53.1% (42.8–63.1%)	98.8% (95.2–99.8%)	96.3% (86.2–99.4%)	78.0% (71.6–83.3%)
IDSA (2013)	71 (72.4%)	27 (27.6%)	/	143 (86.7%)	22 (13.3%)	/	72.4% (62.3–80.8%)	86.7% (80.3–91.3%)	76.3% (66.2–84.3%)	84.1% (77.6–89.1%)

We also validated the diagnosis of PJI cohort and aseptic cohort with ICM criteria. Fifty-two (53.1%) patients in the PJI cohort were correctly diagnosed of infected case and the remaining 46 (46.9%) patients were ignored by the ICM criteria. One hundred sixty-three (98.8%) patients in the aseptic group were correctly regarded as not infected, and 2 (1.2%) were falsely diagnosed as PJI cases. The overall sensitivity and specificity of the ICM criteria were respectively 53.1% (95% confidence interval [CI] 42.8–63.1%) and 98.8% (95% [CI] 95.2–99.8%). (Table 3).

According to the IDSA criteria, 71 (72.4%) patients in the PJI cohort were correctly diagnosed of infected case and the remaining 27 (27.6%) patients were falsely diagnosed as aseptic revision. In the aseptic group, 143 (86.7%) patients were diagnosed as not infected and 22 (13.3%) patients were falsely diagnosed as PJI. The overall sensitivity and specificity of the IDSA criteria were respectively 72.4% (95% confidence interval [CI] 62.3–80.8%) and 86.7% (95% [CI] 80.3–91.3%). (Table 3).

The positive predictive value of the new criteria was not fundamentally different from that of ICM (96.3%, 95% CI 86.2–99.4%) but well ahead of IDSA (76.3%, 95% CI 66.2–84.3%) criteria. The negative predictive value of the new criteria was far better than that of ICM (78.0%, 95% CI 71.6–83.3%) and IDSA (84.1%, 95% CI 77.6–89.1%) (Table 3).

## Discussion

### Diagnosis of PJI

Periprosthetic joint infection (PJI) is a devastating postoperative complication following total joint arthroplasty [14, 15]. Improper diagnosis of PJI may lead to waste of medical resource and delay of the illness. However, the diagnosis of PJI was always controversial in recent years with the further understanding of PJI and new examination continuously applied in the clinical practice [16–18]. Legout and Senneville reported in 2013 that the diagnosis of infection was evoked on a combination of clinical, histological and biopsy, or intraoperative microbiological criteria, but there are no uniform criteria for the definition of PJI [19].

### New definition

The diagnostic criteria of PJI were continually revised, and now, the well-established criteria were raised by ICM and IDSA. The criteria by ICM or IDSA take various effective diagnostic methods into account so that they can correctly diagnose most PJI. However, there is still some limitation for ICM and IDSA [20]. The judgment for every criteria relies on clinicians’ clinical experience, and clinicians may have different views for the same clinical situation.

Therefore, we had better quantify the degree of infection with score and a mature score system can standardize the diagnosis from different clinicians. And more importantly, it is necessary for the new criteria to consist of valuable diagnostic methods as much as possible. The new criteria put forward in 2018 are comprehensive and practical in consideration of these two advantages. The validation group in the original research demonstrated that the new criteria had higher sensitivity and specificity than traditional criteria [13]. However, whether the new criteria can work in wider group of people, such as the Chinese population, remains to be seen. Different race, nationality, and lifestyle may have a profound impact on the diagnosis of PJI. Hence, the objective of this study is to find out if the new criteria are suitable for the Chinese population.

### Chinese population

Patients were divided into two groups according to the established standard, and every patient was scored with the new criteria. The aggregated score consists of preoperative score and intraoperative score. According to the aggregated score, every patient was diagnosed as infected, not infected, or inconclusive. The obtained data was subjected with rigorous and appropriate statistical analysis. For patients in our hospital, the overall sensitivity and specificity of the new criteria were respectively 94.9% (95% confidence interval [CI] 87.9–98.1%) and 95.2% (95% CI 90.3–97.7%). The overall sensitivity and specificity of the ICM criteria were respectively 53.1% (95% confidence interval [CI] 42.8–63.1%) and 98.8% (95% [CI] 95.2–99.8%). The overall sensitivity and specificity of the IDSA criteria were respectively 72.4% (95% confidence interval [CI] 62.3–80.8%) and 86.7% (95% [CI] 80.3–91.3%). In conclusion, the new scoring system really demonstrated a higher sensitivity than ICM and IDSA criteria, and the diagnostic sensitivity could increase substantially by using the new criteria. The specificity of new system was similar to ICM and higher than the IDSA definition. Synthesizing the above statistical results, we believe that there is an enormous advantage for clinicians to choose new diagnostic criteria especially in the Chinese population.

### Limitations

The current study had several limitations that should be acknowledged. One limitation of the study was partially missing data. There was no synovial fluid in some patients’ joint which led to the absence of synovial index data for some patients. Nevertheless, the missing data of synovial index due to dry tap was allowed according to the scoring table of the new definition [13]. Some laboratory indexes, such as synovial alpha-defensin and synovial CRP, were not the routine examination in our hospital, and this part of data was missing. Some diagnostic tests were applied selectively for diagnosis of PJI, for instance, D-dimer, synovial LE, and synovial PMN%. Therefore, this information of some patients was missing especially in the aseptic group. Besides these, partial lost information could also be attributed to incomplete medical records. We excluded the cases with too much information lost and kept data of cases as complete as possible. However, some information was still inevitably lost, and they will lead to bias certainly. On the other side, new criteria demonstrated a greater diagnostic efficiency with the same incomplete data than ICM and IDSA criteria. Second, the patients in the PJI and aseptic groups were filtrated by major criteria and the selection of cases may be subjective and inaccurate. Third, the study was retrospective in nature, with limitations inherent to such a study design. Fourth, we need more study population and longer follow-ups to support our conclusion. The next step of our plan is to conduct a prospective study that could verify the new definition more exactly.

## Conclusions

In conclusion, the 2018 new scoring system put forward by researchers could also apply to the Chinese population for diagnosing PJI following THA or TKA. It demonstrated a higher sensitivity than the ICM and IDSA criteria for patients in the Chinese PLA General Hospital, and the specificity of the new system was similar to existing criteria. Therefore, the new criteria can obviously improve the diagnostic efficiency for PJI compared with the ICM and IDSA criteria. We believe a wider group of people in China could benefit from the new diagnostic criteria and the new definition could provide fresh ideas for improving diagnostic criteria.

## Abbreviations

CRP: C-reactive protein
ESR: Erythrocyte sedimentation rate
ICM: International Consensus Meeting
IDSA: Infectious Diseases Society
MSIS: Musculoskeletal Infection Society
PJI: Periprosthetic joint infection
PMN%: Polymorphonuclear neutrophil percentage
TJA: Total joint arthroplasty
TKA: Total knee arthroplasty
WBC: White blood cell

## Authors’ information

Jiying Chen, Haitao Guan, Jun Fu, Libo Hao, Wei Chai, Xiang Li, and Rui Li come from the Department of Orthopedics, Chinese People’s Liberation Army General Hospital (301 Hospital), 28 Fuxing Rd., 100853, Beijing, PR China. Jing Zhao comes from the Anesthesia/Surgery Center of 301 Hospital.
