# Correction to: The 2018 new definition of periprosthetic joint infection improves the diagnostic efficiency in the Chinese population

**DOI:** 10.1186/s13018-019-1396-2

**Published:** 2019-10-28

**Authors:** Haitao Guan, Jun Fu, Xiang Li, Wei Chai, Libo Hao, Rui Li, Jing Zhao, Jiying Chen

**Affiliations:** 1 Department of Orthopaedics, Chinese People’s Liberation Army General Hospital (301 Hospital), Beijing, People’s Republic of China; 2 Anesthesia/Surgery Center, Chinese People’s Liberation Army General Hospital (301 Hospital), Beijing, People’s Republic of China; 3 School of Medicine, Nankai University, Tianjin, 300071 People’s Republic of China

**Correction to: J Orthopaedic Surg Res**

**http://dx.doi.org/10.1186/s13018-019-1185-y**

In the original publication of this article, [1] the affiliation of the first author Haitao Guan needs to be revised:

Haitao Guan^1,3†^.

^1^Department of Orthopaedics, Chinese People’s Liberation Army General Hospital (301 Hospital), Beijing, People’s Republic of China.

^3^School of Medicine, Nankai University, Tianjin 300,071, People’s Republic of China.
